# Modular ssDNA binding and inhibition of telomerase activity by designer PPR proteins

**DOI:** 10.1038/s41467-018-04388-1

**Published:** 2018-06-07

**Authors:** Henrik Spåhr, Tiongsun Chia, James P. Lingford, Stefan J. Siira, Scott B. Cohen, Aleksandra Filipovska, Oliver Rackham

**Affiliations:** 1 Department of Mitochondrial Biology, Max Planck Institute for Biology of Ageing, D-50931 Cologne, Germany; 2 Harry Perkins Institute of Medical Research and Centre for Medical Research, The University of Western Australia, Nedlands, WA 6009 Australia; 3 Children’s Medical Research Institute, University of Sydney, 214 Hawkesbury Road, Westmead, NSW 2145 Australia; 4 School of Molecular Sciences, The University of Western Australia, Crawley, WA 6009 Australia

## Abstract

DNA is typically found as a double helix, however it must be separated into single strands during all phases of DNA metabolism; including transcription, replication, recombination and repair. Although recent breakthroughs have enabled the design of modular RNA- and double-stranded DNA-binding proteins, there are currently no tools available to manipulate single-stranded DNA (ssDNA). Here we show that artificial pentatricopeptide repeat (PPR) proteins can be programmed for sequence-specific ssDNA binding. Interactions occur using the same code and specificity as for RNA binding. We solve the structures of DNA-bound and apo proteins revealing the basis for ssDNA binding and how hydrogen bond rearrangements enable the PPR structure to envelope its ssDNA target. Finally, we show that engineered PPRs can be designed to bind telomeric ssDNA and can block telomerase activity. The modular mode of ssDNA binding by PPR proteins provides tools to target ssDNA and to understand its importance in cells.

## Abstract (web summary)

Pentatricopeptide repeat proteins bind single-stranded RNA and have been used to study ssRNA biology. Here the authors co-opt these proteins to target ssDNA and demonstrate specific binding of telomere sequences, the structural basis for ssDNA wrapping, and use them as potent telomerase inhibitors.

## Introduction

DNA is predominantly found as a stable duplex in biological systems. However, accessing the genetic information stored in DNA for transcription, replication, recombination and repair requires the separation of DNA duplexes and exposure of single-stranded DNA (ssDNA). The presence of ssDNA also ensures the recruitment of the enzymatic activities required for these processes^1^. On the other hand, exposed ssDNA is particularly susceptible to both chemical and enzymatic damage and cells have a variety of mechanisms to protect ssDNA and ensure the rapid return of ssDNA to a duplex state^2^. If these mechanisms fail there are severe consequences for the cell, such as replication stalling that results in rapid increases in ssDNA. This exposed ssDNA acts as a marker of stress and activates cell signalling pathways to halt cell cycle progression and mobilise DNA repair processes and, if these fail, initiation of cell destruction via apoptosis^3^. Interestingly, despite its vulnerability, a large number of viruses and bacteriophages use ssDNA as the transmissible form of their genomes. The mutation rates of ssDNA viruses are extremely high and approach those of viruses with RNA genomes^4^. This might provide an evolutionary advantage, enabling a pool of viruses with variant genomes to be produced from each infected cell, some of which might have a selective advantage in subsequent infections. In another important biological process, bacterial conjugation, ssDNA plays an important role as it is the form by which genetic information is transferred to recipient cells^5^. In addition, ssDNA plays an important role in the maintenance and function of telomeres^6^.

Telomeres protect the ends of linear eukaryotic chromosomes from degradation and from fusion with other chromosomes^6^. The repetitive telomeric sequences consist of long double-stranded G-rich sequences followed by short, 50–150-nucleotide, single-stranded ends synthesised by the telomerase reverse transcriptase^7–11^. In humans, telomeres are usually protected by a six-protein complex, known as shelterin^8, 12^. Of the six shelterin proteins, only Pot1 binds single-stranded telomeric DNA^13^. Although robust technologies have now emerged that enable the site-specific manipulation of dsDNA and RNA in living cells^14–16^, the manipulation of ssDNA has not been possible to date. Because natural ssDNA-binding proteins use binding domains that recognise a combination of sequence and structure, for example, OB folds and RNA recognition motifs^1^, they cannot be easily repurposed to bind new ssDNA targets. Since ssDNA is very similar in structure to ssRNA we tested whether ssRNA-binding proteins that have been discovered to have modular recognition properties could be adapted for programmable ssDNA binding.

PPR proteins are RNA-binding proteins found predominantly in mitochondria and chloroplasts^17^. They regulate RNA processing, translation, stability and editing, and possess well-characterised, modular RNA recognition properties^17–20^. PPRs typically consist of tandem arrays of 2–30 degenerative repeats and each repeat usually consists of 35 amino acids. PPRs stack on each other to form an extended solenoid structure that can recognise RNA in a sequence-specific manner based on the identities of amino acids at positions 5 and 35 of each repeat^21–24^. Due to the highly insoluble nature of PPR proteins it has been difficult to understand their structural and RNA-binding properties. To bypass these limitations, we previously used consensus design to engineer artificial PPR domains (cPPRs) that are highly soluble and, via an appropriate choice of amino acids at position 5 and 35, can be designed to bind RNA in a predictable, sequence-specific manner^21^. These engineered PPR scaffolds enable the predictable binding of RNA targets and provide a starting point to use engineered PPR proteins to rationally manipulate cellular gene expression. In this study we have discovered that cPPRs bind not only RNA but also ssDNA in a modular and programmable manner, analogous to their RNA-binding capability. Crystal structures of cPPRs with and without a ssDNA target provide an explanation for their ability to bind ssDNA and show how rearrangements of hydrogen bonds within and between protein repeats enable them to wrap around their targets. We re-design cPPRs to bind telomeric ssDNA and demonstrate that they can inhibit the activity of human telomerase in vitro. Unlike natural Pot1 that binds rigidly to its designated target, cPPRs can be programmed to bind any telomere sequence of interest. This opens up new opportunities to engineer artificial telomere-protective proteins and to understand fundamental aspects of telomere biology.

## Results

### Modular ssDNA binding by consensus PPR proteins

**Fig. 1:**
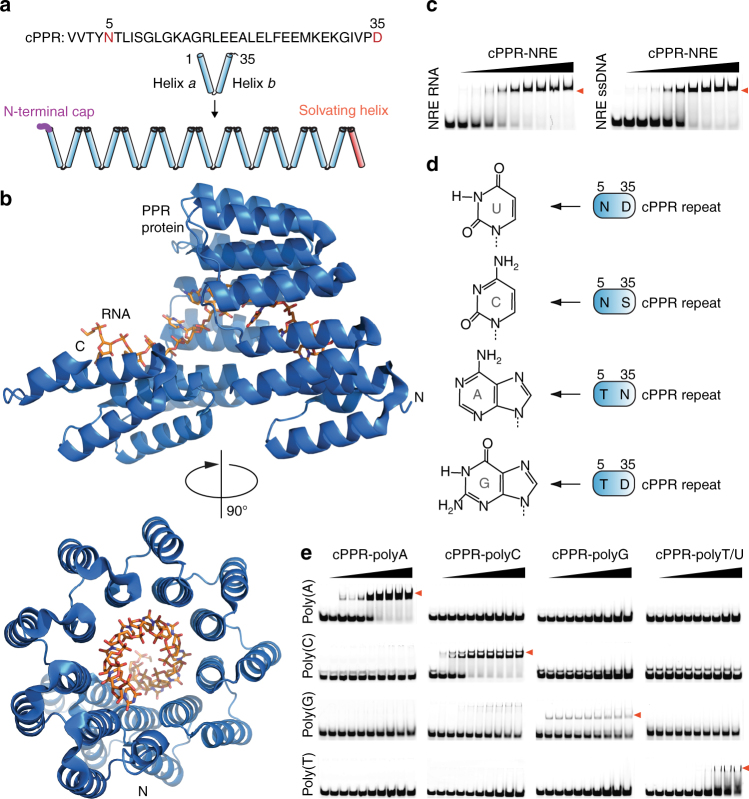
Consensus pentatricopeptide repeat (cPPR) proteins bind ssDNA. **a** The consensus PPR sequence and its assembly into a repeat protein flanked by stabilising elements. Annotation of the PPR sequence is based on the numbering scheme of Yin et al.^34^, where residues 5 and 35 are equivalent to residues 4 and ii according to Kobayashi et al.^51^, or 4 and 34 according to Fujii et al.^52^. **b** Crystal structure of a designer PPR protein bound to its RNA target. Structure determined by Shen et al.^24^. **c** Electrophoretic mobility shift assays (EMSA) of the cPPR-NRE using RNA and single-stranded DNA (ssDNA) Nanos response element (NRE) probes. Bound complexes are highlighted with red arrows. Protein concentrations used were, from left to right: 0, 0.15, 0.3, 0.5, 1, 2, 4, 6, 8 and 10 µM. **d** The modular PPR code for RNA binding. Amino acids at positions 5 and 35 of each cPPR repeat recognise specific RNA bases. **e** Purified proteins were titrated against homopolymeric ssDNA probes in a DNA EMSA. Complexes formed between predicted cognate ssDNA–protein pairs are indicated with red arrows and demonstrate that high specificity of each cPPR protein for its cognate ssDNA target. Protein concentrations used were, from left to right: 0, 0.15, 0.3, 0.5, 1, 2, 4, 6, 8 and 10 µM

Although PPRs are regarded as RNA-binding motifs, there is little information on their nucleic acid specificity. The plant PPR protein OTP87 was reported to bind ssRNA but not ssDNA, dsDNA or dsRNA^25^, and the plant THA8 protein was reported to bind ssRNA with >100-fold higher affinity than ssDNA^26^. However, detailed comparisons have been hampered because of PPR proteins’ instability and insolubility. Because our synthetic consensus PPR (cPPR) proteins have very robust RNA-binding properties and stabilities^21^, we used these as a basis to examine nucleic acid-binding specificity. Typical cPPRs consist of eight identical synthetic repeats flanked by an N-terminal cap consisting of Met-Gly-Asn-Ser, because statistically, Gly, Asn and Ser have the highest propensities to occur at these N-terminal positions in α helices (Fig. 1a and Supplementary Fig. 1)^27^. In addition, a C-terminal solvating helix is added after the final consensus repeat to prevent unfolding, according to the successful consensus tetratricopeptide repeat domain design of Main et al.^28^.

Synthetic PPR proteins fold into solenoid structures like natural PPR proteins^21, 29^ and a recent study from Shen et al.^24^ showed that they wrap around their RNA targets (Fig. 1b), however a molecular explanation for any potential discrimination between RNA and ssDNA is lacking. We initially tested a cPPR protein designed to bind a naturally occurring RNA sequence known as the Nanos response element (NRE). We found that this cPPR bound tightly to a ssDNA NRE oligonucleotide as well as to an RNA NRE oligonucleotide (Fig. 1c). Surprisingly, the affinity was only ~3-fold reduced between ssDNA and RNA (Fig. 1c and Supplementary Fig. 2). Based on bioinformatics predictions^22, 23^ we previously identified particular amino acids at positions 5 and 35 of our cPPR that enable the specific recognition of individual RNA bases^21^. Although the NRE-targeting cPPR bound ssDNA, it was not clear if it bound the ssDNA specifically according to the PPR code. Therefore, we produced four different cPPR proteins designed to bind homopolymers of adenine, cytosine, guanine or uracil and tested their binding using ssDNA homopolymers (Fig. 1d). We found that the binding of ssDNA homopolymers conformed to the specific code for base recognition established previously for RNA (Fig. 1e). The binding of poly(A) ssDNA was slightly reduced compared to poly(A) RNA, while binding to poly(C) ssDNA was actually slightly better than for poly(C) RNA (Supplementary Fig. 2). Exact binding affinities for poly(G) ssDNA and RNA were difficult to determine, likely due to the propensity of poly(G) tracts to form G-quadruplex structures. Interestingly, binding to poly(T) ssDNA was greatly reduced compared to poly(U), possibly due to the additional methyl group on the thymine base relative to uracil. Therefore, the code for base recognition by PPRs is maintained when binding ssDNA targets, opening the way for the use of cPPRs to target ssDNAs in a programmable manner.

### Inhibition of human telomerase by designed cPPR proteins

**Fig. 2:**
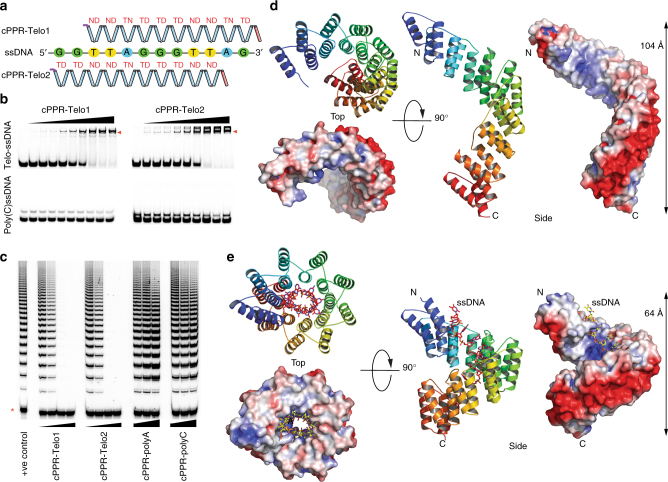
cPPRs designed to bind to telomeric sequences. **a** Cartoon depicting the design and binding sites of cPPR-Telo1 and cPPR-Telo2. The amino acids at positions 5 and 35 of each repeat in the 10-repeat cPPRs are shown in red. The ssDNA probe is derived from the repeating telomeric DNA sequence and contains the binding sites for both cPPR-Telo1 and cPPR-Telo2. **b** ssDNA EMSA using Telo-ssDNA or poly(C) ssDNA (control). ssDNA–protein complexes are highlighted with red arrows. Protein concentrations used were, from left to right: 0, 0.15, 0.3, 0.5, 1, 2, 4, 6, 8 and 10 µM. **c** Inhibition of telomerase extension by telomere-targeting cPPRs in telomerase activity assays with immunopurified telomerase and a primer incorporating 18 nt of telomeric sequence. Increasing concentrations of cPPR-Telo1 and cPPR-Telo2 (50, 100, 200 and 300 nM) were added to the indicated reactions and cPPR-polyA and cPPR-polyC (300 nM, 1 µM and 3 µM) were used as control proteins. The red asterisk indicates a 30-mer 5′-^32^P-labelled recovery/loading control. **d** Overall structure of cPPR-Telo1, shown in cartoon representation coloured in rainbow colours with blue at the N terminus and red at the C terminus. The surface charge distribution is coloured according to the local electrostatic potential (blue, +5 kT; red −5 kT). **e** Structure of cPPR-Telo1 in complex with DNA, which is shown as orange or yellow sticks

We examined if cPPRs could be designed to target biologically relevant ssDNA targets and focused on the repeating telomeric ssDNA sequences of mammalian cells. We designed two cPPRs targeting staggered sequences within the telomeric repeats (Fig. 2a and Supplementary Fig. 1b). In analogy to Pot1^30^, we choose to build cPPRs consisting of 10 repeats to match the length of the Pot1 recognition sequence. Both telomere-targeting cPPRs, dubbed cPPR-Telo1 and cPPR-Telo2, bound telomeric ssDNA sequences with high affinity and specificity, as determined by binding assays with off-target ssDNAs (Fig. 2b and Supplementary Fig. 3). Binding to ssDNA was robust in the presence of variable pH, salt concentrations and in the presence of non-specific competitor ssDNA (Supplementary Fig. 4). Furthermore, we compared the ability of cPPR-Telo1 and cPPR-Telo2 to bind ssDNA in G-quadruplex structures by comparing binding to two targets, where one could form a G-quadruplex structure and the other could not^31^. Both designed proteins could bind efficiently independent of potential formation of a G-quadruplex (Supplementary Fig. 5), which is important since telomeric ssDNA is well known for its ability to form robust G-quadruplexes. We used the extension of telomeric ssDNA by telomerase as a model system to examine whether cPPRs could modulate ssDNA function. We performed direct telomerase extension assays^32^ using human telomerase over-expressed and assembled in HEK-293T cells and examined whether the specific binding of the telomeric ssDNA by cPPR-Telo1 and cPPR-Telo2 could modulate telomerase activity. We found that both designed cPPRs effectively blocked telomerase activity in a dose-dependent manner, while control cPPR proteins had no effect (Fig. 2c). Furthermore, inhibition of telomerase extension was maintained even when non-specific competitor ssDNA was present in orders of magnitude excess (Supplementary Fig. 6).

The position of the Pot1-binding site relative to the 3′-end of the ssDNA template has been shown to have a dramatic effect on telomerase activity, with 3′-end binding blocking telomerase extension and internal binding stimulating the processivity of telomerase^33^. To examine whether these effects were an inherent property of Pot1 or resulted simply from the locations of protein binding we tested cPPR-Telo2’s ability to modulate the extension of telomerase on a variety of different primers where the binding locations were at varied distances from the 3′-end of the ssDNA. We found that cPPR-Telo2 blocked telomerase activity regardless of its binding location, indicating that the multiple regulatory modes of Pot1 are idiosyncratic to that particular protein (Supplementary Fig. 7a). The potent inhibition of telomerase likely results from the cPPR’s ability to block access of telomerase to ssDNA, since we found that cPPR-Telo2 could protect its ssDNA target from DNase I digestion (Supplementary Fig. 7b).

### Structure of a telomeric ssDNA-bound cPPR protein

Next, we sought to obtain structural information on the two cPPR proteins specifically designed to bind to telomeric ssDNA sequences, to understand further the cPPR scaffold’s ability to bind both ssDNA and RNA. Although these proteins are highly similar in their primary sequences, only cPPR-Telo1 gave reproducible and diffracting crystals. We solved the structure of cPPR-Telo1 and cPPR-Telo1 bound to its ssDNA target using single-wavelength anomalous dispersion at 2.1 and 2.0 Å resolution, respectively (Table 1, with sample electron densities in Supplementary Fig. 8). The cPPR-Telo1 protein adopted an overall fold that closely mimicked other designed PPR structures^21, 24, 29^ (Fig. 2d, e), where the two helices of each repeat, helix *a* and helix *b*, form a hairpin and these hairpins stack upon each other to form a right-handed superhelix. Mapping the electrostatic potential revealed a highly asymmetrical surface charge distribution with positively charged residues in the ssDNA-binding cavity and a negatively charged band along the exterior, exposed face of the superhelix (Fig. 2d, e). This charge distribution might provide the initial driving force for nucleic acids to bind the PPR proteins, based on their negatively charged phosphate backbones.

**Table 1:** Data collection and refinement statistics

	apo cPPR-Telo1	cPPR-Telo1/ssDNA
*Data collection*		
Space group	*P*2_1_2_1_2_1_	*P*4_1_2_1_2
Cell dimensions		
* a*, *b*, *c* (Å)	86.4, 87.1, 91.6	114.8, 114.8, 83.5
*α*, *β*, *γ* (°)	90, 90, 90	90, 90, 90
Resolution (Å)	19.70–2.08 (2.15–2.08)	27.41–1.95 (2.02–1.95)
*R*_merge_	0.111 (1.306)	0.0714 (1.823)
CC_1/2_	0.998 (0.719)	0.999 (0.742)
*I* /*σ*(*I*)	9.92 (1.24)	30.79 (2.23)
Completeness (%)	96.62 (81.79)	99.21 (99.63)
Redundancy	10.5 (4.9)	27.1 (28.7)
*Refinement*		
Resolution (Å)	19.70–2.08 (2.13–2.08)	27.41–1.95 (2.0–1.95)
No. of reflections	40,891	40,921
*R*_work_/*R*_free_	0.240/0.280	0.189/0.228
No. of atoms		
Protein	2683	2785
DNA		210
Water	840	377
*B*-factors		
Protein	26.10	29.27
DNA		19.03
Water	44.88	41.41
R.m.s. deviations		
Bond lengths (Å)	0.014	0.010
Bond angles (°)	1.67	1.01
Ramachandran plot favoured/allowed (%)^a^	99.71/0.29	98.07/1.93
Clashscore^a^	2.93	3.17
Molprobity score^a^	1.58	1.5

Statistics for the highest-resolution shell are shown in parentheses

^a^According to the definition used in Molprobity^44^

**Fig. 3:**
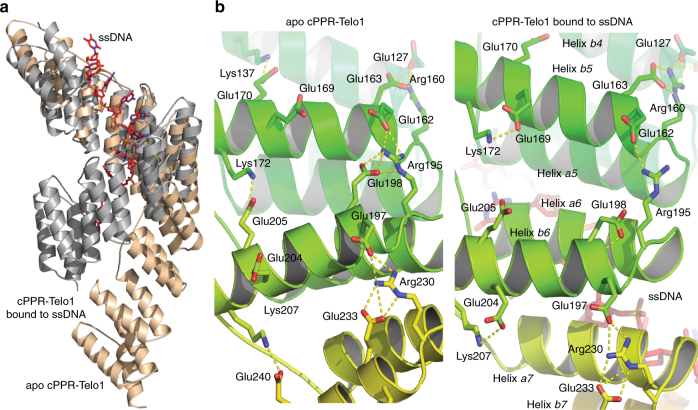
An altered hydrogen bond network enables compaction of PPRs upon ssDNA binding. **a** Structural comparison of apo cPPR-Telo1 (wheat) and in complex with ssDNA (grey). **b** Altered hydrogen bond network between *b* helices of cPPR-Telo1 upon ssDNA binding. Amino acids participating in hydrogen bond rearrangements are shown as sticks and hydrogen bonds are shown with dashes. Arg160, Arg195 and Arg230 correspond to Arg16 in the standardised annotation of PPR motifs based on the numbering scheme of Yin et al.^34^; Glu162 and Glu197 to Glu18; Glu163, Glu198 and Glu233 to Glu19; Glu169 and Glu204 to Glu25; Lys137, Lys172 and Lys 207 to Lys28; and Glu170, Glu205 and Glu240 to Glu29

Our structures provide the first pair of structures where an identical PPR protein compacts upon binding a nucleic acid in the canonical, sequence-specific mode, providing an opportunity to understand how the conformation of the protein changes upon ligand binding. cPPR-Telo1 wraps around the ssDNA upon binding, inducing a massive compaction of the superhelical structure and decreasing the length of the protein from 104 to 64 Å (Figs. 2d, e and 3a). This conformational change more than halves the number of cPPR protein side chains exposed to water and may be the principal driving force favouring nucleic acid binding. Overall, the rearrangement is based on the *a* helices on the concave side of the protein moving into closer proximity as bonds are formed to the DNA with a concomitant separation of the *b* helices on the convex side. To allow the separation, an extensive hydrogen bond network between an arginine at position 16 and a glutamic acid at position 19 from one PPR repeat and a glutamic acid at position 18 from the preceding repeat in the apo protein rearranges at three out of nine possible positions leading to a more open arrangement when bound to DNA (Fig. 3b). Specifically, the side chains of Glu128, Glu198 and Glu303 form hydrogen bonds to their own main chain instead of to Arg125, Arg195 and Arg300, respectively. In addition, a hydrogen bond between a lysine at position 28 in each repeat and a glutamic acid at position 26 of the succeeding repeat switches to an intra-helix hydrogen bond interaction between this lysine and a nearby glutamic acid at position 25 (Fig. 3b). These and other changes in van der Waals interactions produce conformational variations that are amplified over the entire cPPR, resulting in the notable compression of the protein superhelix.

### Interactions between cPPR-Telo1 and ssDNA

**Fig. 4:**
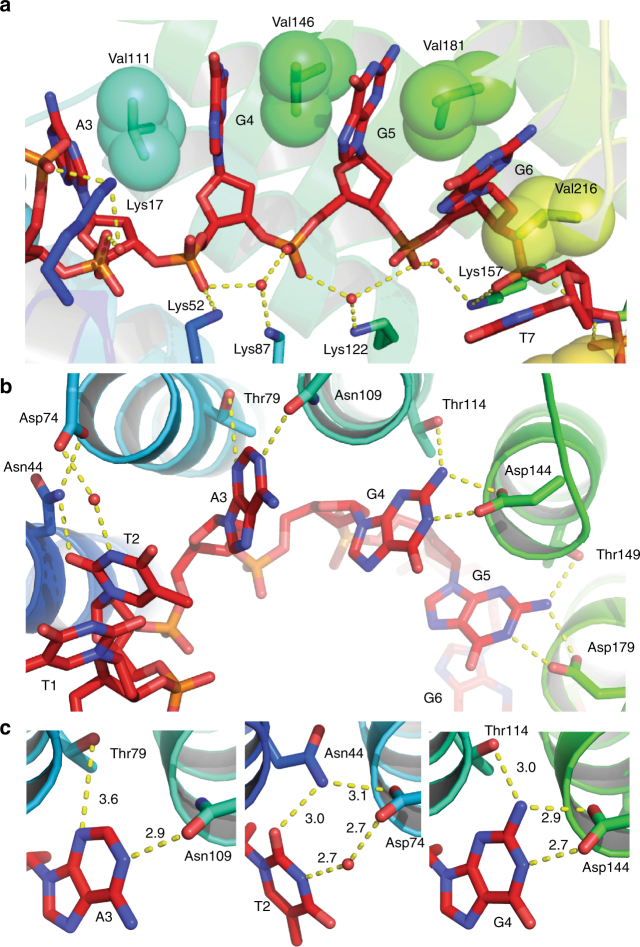
Protein–ssDNA interactions of cPPR-Telo1. **a** Non-specific protein–DNA interactions. Stacking valines are shown as sticks and spheres, and interacting polar residues and ssDNA are shown as sticks. Hydrogen bonds are shown with dashes. **b** Specific protein–ssDNA interactions. **c** Nucleotide base recognition by cPPR-Telo1 according to the PPR code. Interacting polar residues of cPPR-Telo1 and adenine (A), thymine (T) and guanine (G) are shown as sticks. Hydrogen bonds are shown with dashes and distances in Å are indicated

Specific recognition of RNA by PPR proteins has been found to occur via hydrogen bonding between the side chains of residues at positions 5 and 35 of each PPR motif and the Watson–Crick faces of the nucleotide bases^34^. The amino acid at position 5 forms direct hydrogen bonds with the RNA base, while the amino acid at position 35 directly bonds with purine bases but makes a water-mediated interaction in the case of pyrimidines. We observed the same binding mode in our cPPR-ssDNA structure (Fig. 4) and likewise the bases were sandwiched between the valine residues at position 2 of each repeat (Fig. 4a). Furthermore, the phosphate backbone of the ssDNA was bound by lysine residues at position 13 of each repeat (Fig. 4a), as we^21^ and others^24^ had observed previously for RNA. The conserved modes of cPPR-base recognition between RNA and ssDNA are reflected in the cPPR’s ability to bind both ssDNA and RNA (Fig. 1c, e). Interestingly, the hydrogen bond between the threonine at position 5 and adenine is rather long (3.6–3.9 Å), compared to when threonine in this position binds guanine (2.9–3.1 Å), which appears to be more ideal (Fig. 4c). This phenomenon is also true in the structures of designed PPR proteins in complex with RNA^24^ but if another residue at position 5 might be more efficient in the binding of adenine, or whether this arrangement is advantageous for some reason remains to be determined.

In the case of uracil recognition in PPR-RNA complexes, the amide group of the asparagine side chain at position 5 donates a hydrogen bond to the O2 of the pyrimidine ring, while the carboxyl group of the aspartate makes a water-mediated hydrogen bond with the N3 of the pyrimidine^34^. An identical mode of thymine recognition is seen in our structure when ssDNA is bound and the methyl group at position C5 that distinguishes uracil and thymine is orientated away from the protein (Fig. 4a–c), such that it is initially hard to predict why poly(T) ssDNA is bound much less efficiently than poly(U) RNA, based on this difference. However, the C5 methyl groups likely alter the hydration pattern of the backbone and could also alter the stacking energies between bases, and thereby affect the dynamics of the RNA–protein interaction^35^. Support for this hypothesis comes from the observation that although the electron density is very clear for all nucleotides, it is weaker for one of the thymines (T7) in the structure (Supplementary Fig. 9). T7 sits adjacent to another thymine (T8) and the altered stacking energy between these bases might contribute to the observed flexibility at that position. Although tolerated well, or to some extent, when there is an isolated thymine or a pair of thymines in the ssDNA target, respectively, it could be that the additive disruption in stacking energies causes poly(T) tracts to bind poorly to cPPR proteins (as seen in Fig. 1e and Supplementary Fig. 2).

**Fig. 5:**
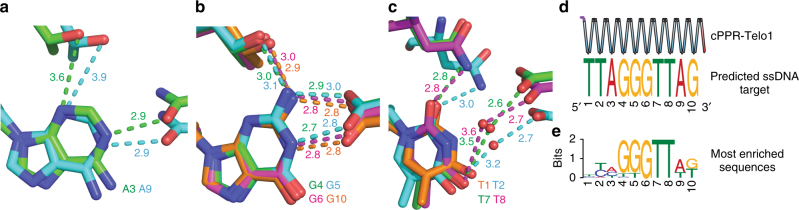
Recognition of ssDNA bases across the length of the artificial PPR protein. **a** Recognition of adenines at positions 3 and 9. **b** Recognition of guanines at positions 4, 5, 6 and 10. **c** Recognition of thymines at positions 1, 2, 7 and 8. Hydrogen bonds are shown with dashes and distances in Å are indicated. **d** Bind-n-Seq analysis of cPPR-Telo1 protein ssDNA-binding specificity, compared to its designed target. **e** A logo representing the most enriched sequences (1127 in total, all with *E*-values > 8.6e-18) found in the cPPR-Telo1-bound ssDNA library, as determined by discriminative regular expression motif elicitation (DREME)^50^. No significantly enriched motifs were found in a matched unselected library

Comparing the recognition of bases by each of the 10 PPRs revealed that the hydrogen bonding was very similar within each type of base. This included the long hydrogen bond between the threonine at position 5 and adenine (Fig. 5a), the compact hydrogen bonding network recognising guanine (Fig. 5b) and the water-mediated hydrogen bonds recognising thymines (Fig. 5c). One interesting exception is the thymine at position 1, which does not make any specific hydrogen bonds with the protein in our structure. To further examine this observation and the specificity of base recognition at all positions in cPPR-Telo1 we performed a comprehensive Bind-n-Seq analysis using a randomised ssDNA target sequence (Fig. 5d, e). We found significant enrichment of the predicted base at each position in the selected library, further demonstrating the specificity of cPPRs for ssDNA, however the enrichment of thymine at position 1 was less than the other positions. Therefore, the thymine at position 1 might be in a dynamic equilibrium between bound and unbound states. A recent examination of the binding of RNA to designer PPR proteins found that the terminal positions in the target nucleic acids made the least contribution to the binding affinities of these complexes^36^. Our data confirm this observation in the context of ssDNA and also provide structural evidence for this effect.

## Discussion

Here we found that designed PPR proteins can bind ssDNA in a sequence-specific manner analogous to how they bind RNA. Furthermore, the modularity of the cPPR proteins enabled the design of cPPRs that could target telomeric ssDNA and block extension by human telomerase. The ability of cPPRs to target ssDNA begs the question: do natural PPR proteins generally discriminate between RNA and DNA? The 2′-OH groups in the bound RNA are oriented towards helix *a* and discrimination between ssDNA and RNA might be achieved by interactions with the side chains of residues at positions 6 and 9 of helix *a*. However, identifying the exact residues involved is complicated by the fact that PPR arrays compress significantly upon RNA binding^34, 37^. This reorientates the helices so different residues might be involved in initial recognition of the backbone sugar, compared to those involved in later accommodation within the PPR solenoid. The molecular basis for potential discrimination between ssRNA and ssDNA by the wide variety of natural PPR proteins is not clear. The THA8 PPR protein binds its RNA target as a dimer and only a central G is recognised according to the PPR code by a PPR repeat from one of the THA8 subunits^26^. A direct hydrogen bond between an arginine residue at position 8 of that repeat and the 2′-OH group is required for high-affinity RNA binding. The ribose group of the adjacent A nucleotide is also recognised by THA8 but via a water-mediated hydrogen bond with an arginine of the other THA8 protein in the dimer. The PPR10 protein has 19 PPRs and 3 of these interact with the 2′-OH ribose groups in its target RNA: repeats 3 and 5 make direct hydrogen bonds via arginine and serine amino acids at positions 2 and 5, respectively, while repeat 15 makes a water-mediated hydrogen bond, via a serine at position 9^34^. Consistent with these observations we found that recombinant PPR10 did not robustly bind ssDNA, in comparison to RNA (Supplementary Fig. 10). Therefore, the mode of recognition of ribose 2′-OH groups by different PPR proteins appears to be idiosyncratic and the specificity for RNA in natural proteins might be wiped clean in the consensus design of cPPRs, resulting in their ability to bind both RNA and ssDNA with high affinity.

In nature RNA-binding proteins do not often need to discriminate against ssDNA^1^. First, because RNAs are usually orders of magnitude more abundant than the genes that encode them in cells, the DNA can be simply outcompeted by RNA. Second, ssDNA is sequestered in cells by non-specific ssDNA-binding proteins, such as replication protein A (RPA)^2^, limiting the access of RNA-binding proteins. Third, in eukaryotic cells RNA-binding proteins are often targeted to the locations of their RNA targets, such as the cytoplasm, mitochondria and the nucleoli, which are separated from potential ssDNA targets in the nucleus^1^. The cPPR scaffold provides high-affinity, sequence-specific binding to aid in the goal of developing designer ssDNA-binding proteins, and our current study sets the stage for further engineering of cPPRs to discriminate against RNA, to more efficiently target ssDNA in cells. Furthermore, if combined with protein signals to efficiently target cPPRs to regions of the cell containing their ssDNA targets, these proteins could help reveal the many aspects of cell biology involving ssDNA, which are currently understudied due to a lack of appropriate tools.

In this study we provide proof of principle that cPPRs can be used to target and manipulate telomeric ssDNA in vitro. While further work will be required to explore the applications of these proteins in cellular systems, one attractive area in ssDNA biology is to manipulate telomerase and telomeres in general. Telomerase uses a unique mode of primer extension, whereby an internal RNA template is used to specify the sequence of the added repeat^10^. Because this template must be re-used for each repeat, the product-template duplex must dissociate after each addition, providing ample opportunity for the engineered cPPRs to access the telomeric ssDNA. Human telomerase is proposed to extend telomeres by 5–10 repeats per cell division^38^. This may result from the complex competition between telomerase, POT1, the general ssDNA-binding protein RPA, and other shelterin components^39^. The addition of a telomeric sequence-specific cPPR in cells could be useful to skew this competition by blocking telomerase access and preventing telomere extension—to study telomere biology and as potential cancer therapeutics in the future. Overall, the manipulation of ssDNA has been neglected in biological research to date and the telomere is just one of many areas where new tools will be very valuable. In this study we have shown that we have the ability to design proteins that recognise specific ssDNA sequences of interest, with many potential applications in biology and biotechnology.

## Methods

### Design and synthesis of cPPR coding sequences

cPPR-polyA, cPPR-polyC, cPPR-polyG and cPPR-polyU were designed as described in Coquille et al.^21^ and shown in Supplementary Fig. 1a. cPPR-Telo1 and cPPR-Telo2 were designed based on 10 tandem repeats with amino acids at position 5 and 35 chosen according to the recognition code of cPPRs described in this article. N-terminal cap residues (Met-Gly-Asn-Ser) and a C-terminal solvating helix (Val-Thr-Tyr-Thr-Thr-Leu-Ile-Ser-Gly-Leu-Gly-Lys-Ala-Gly) were added to the final design (see Supplementary Fig. 1b for a detailed example). Synthetic genes encoding the final cPPR design were optimised for expression in *Escherichia coli* and synthesised from overlapping oligonucleotides (GeneArt and GenScript).

### Protein purification

Coding sequences for cPPR-Telo1, cPPR-Telo2 and enhanced green fluorescent protein (EGFP) were subcloned into pETM30 and expressed as fusions to glutathione *S*-transferase and His tags in *E. coli* 2566 cells (New England Biolabs). Cells were lyzed by sonication in lysis buffer (50 mM Tris-HCl, pH 8.0, 0.3 M NaCl and 5 mM imidazole). Lysates were clarified by centrifugation and incubated with His Select Beads (Sigma) for 30 min with gentle rocking at 4 °C. Beads were washed twice with wash buffer (same as lysis buffer but with 10 mM imidazole) and transferred into a Poly-Prep Chromatography Column (Bio-Rad). Beads were washed twice with wash buffer and proteins were eluted in elution buffer (same as lysis buffer but with 250 mM imidazole). Purified proteins were dialysed using DiaEasy Dialyzer (BioVision) in dialysis buffer (25 mM Tris, pH 7.4, 0.2 M NaCl, 0.5 mM EDTA and 2 mM dithiothreitol (DTT)) overnight at 4 °C. Protein concentration was determined by the bicinchoninic acid assay using bovine serum albumin (BSA) as a standard. cPPR-polyA, cPPR-polyC, cPPR-polyG, cPPR-polyU/T and cPPR-NRE were purified as described by Coquille et al.^21^. Protein used for crystallisation was essentially purified as above with the following modifications: during dialysis, the fusion tag was removed by tobacco etch virus at a protease:protein molar ratio of 1:50, followed by purification on a Superdex 200 10/300 column in dialysis buffer and concentrated using Vivaspin concentrators (10 000 Da molecular weight cutoff, GE).

### Crystallisation and structure determination

Crystals of cPPR-Telo1 were grown at 23 °C by the sitting drop vapour diffusion method by mixing 1 μl protein (5 mg/ml) with an equal volume of reservoir solution (100 mM sodium acetate (pH 4.6), 20 mM calcium chloride and 40% MPD). In addition, cPPR-Telo1 was incubated with target ssDNA oligonucleotide (TTAGGGTTAG) at a protein/DNA molar ratio of 1:2 for 30 min at 4 °C and crystallised in 100 mM sodium acetate (pH 4.6), 20 mM calcium chloride and 22 % MPD. For both cPPR-Telo1 and cPPR-Telo1/ssDNA a single-wavelength anomalous dispersion data set was collected at the K-edge of selenium at beamline ID30A-3 (ESRF, Grenoble, France). The X-ray diffraction data (Table 1) were processed with X-ray diffraction spectroscopy^40^ and the structure was solved with PHENIX^41^ and autoSHARP^42^. An initial model built by Buccaneer^43^ or Autobuild^41^ was used as a starting model for manual building in COOT^44^ interspersed with refinement in Buster (version 2.10.3)^45^, which rendered a final model (Table 1) that had no Ramachandran outliers as assessed by MOLPROBITY^46^. Representative portions of the electron densities of the final cPPR-Telo1 and cPPR-Telo1/ssDNA models are shown as stereo images in Supplementary Fig. 8. Figures were prepared with PyMOL (Schrödinger) and structural alignments were performed using the secondary-structure matching method.

### Electrophoretic mobility shift assays

Purified proteins were incubated at room temperature for 30 min with 0.83 µM fluorescein-labelled ssDNA or RNA oligonucleotides (Dharmacon) in EMSA buffer (10 mM HEPES, pH 8.0, 1 mM EDTA, 50 mM KCl, 2 mM DTT, 0.1 mg/ml fatty acid-free BSA and 0.02% Tween-20). Sequences for probes are listed below:

NRE RNA: 5′-(Fl)rArUrUrGrUrArUrArUrA-3′;

NRE: 5′-(Fl)AUUGUAUAUA-3′;

Poly(A) RNA: 5′-(Fl)rArArArArArArArArArA-3′;

Poly(C) RNA: 5′-(Fl)rCrCrCrCrCrCrCrCrCrC-3′;

Poly(G) RNA: 5′-(Fl)rGrGrGrGrGrGrGrGrGrG-3′;

Poly(U) RNA: 5′-(Fl)rUrUrUrUrUrUrUrUrUrU-3′;

Poly(A): 5′-(Fl)AAAAAAAAAA-3′;

Poly(C): 5′-(Fl)CCCCCCCCCC-3′;

Poly(G): 5′-(Fl)GGGGGGGGGG-3′;

Poly(T): 5′-(Fl)TTTTTTTTTT-3′;

Telo-ssDNA: 5′-(Fl)GGTTAGGGTTAG-3′;

Telo-ssDNA 1 mismatch: 5′-(Fl)GGTTAGCGTTAG-3′;

Telo-ssDNA 3 mismatches: 5′-(Fl)GGTTACCCTTAG-3′;

Telo-ssDNA 5 mismatches: 5′-(Fl)GGTTCCCCCTAG-3′;

Primer GG-a: 5´-(Fl)GGTTAGGGTTAGGGTTAGGG-3´;

Primer a: 5′-(Fl)TTAGGGTTAGGGTTAGGG-3′, where r designates a ribonucleotide and (Fl) designates fluorescein followed by an 18-atom hexa-ethyleneglycol spacer. A non-specific competitor ssDNA derived from the 15 nt of spacer DNA of the telomerase extension assay substrates with sequence 5′-CTAGACCTGTCATCA-3′ was added where indicated. Reactions were analysed by 10% PAGE in TAE and fluorescence was detected using Typhoon FLA 9500 Biomolecular Imager (GE). Assays were replicated at least three times.

### Immunopurification of telomerase

Purification of human telomerase was performed as described in Tomlinson et al.^47^. Briefly, an affinity purified sheep polyclonal anti-hTERT antibody (20 µg) was added per ml of cleared lysate from HEK-293T cells and rotated for 1 h at 4 °C. Protein G-agarose beads (50 µl of a 50% vol/vol slurry per ml lysate) were added and the suspension rotated for 1 h at 4 °C. The protein G suspension was collected in a 15 mm ID × 200 mm L fritted glass column (Bio-Rad), and washed with five column volumes of lysis buffer without DTT and phenylmethylsulfonyl fluoride. To elute the purified enzyme, 100 µl of 1 µM antigenic peptide (ARPAEEATSLEGALSGTRH) in storage buffer (20 mM HEPES-KOH (pH 7.9), 2 mM MgCl_2_, 300 mM KCl, 1 mM DTT, 10% v/v glycerol and 0.1% v/v Triton X-100) was added per ml of lysate and incubated with shaking at room temperature for 1 h. The eluate was collected into a Protein Lo-Bind tube (Eppendorf), snap-frozen in liquid nitrogen and stored at −80 °C. The yield of immunopurified telomerase enzyme complex was determined by dot-blotting against hTR, using the DNA oligonucleotide probe 5′-32P-CGGTGGAAGGCGGCAGGCCGAGGC-3′ for detection^32, 47^.

### Direct primer extension activity assay

Telomerase activity assays were performed as described in Tomlinson et al.^47^. Briefly, immunopurified telomerase was incubated alone or in combination with cPPR-Telo1, cPPR-Telo2 or EGFP proteins. Telomerase extension assay was performed at 37 °C for 4 h in 20 mM HEPES-KOH buffer, pH 8, 150 mM KCl, 2 mM MgCl_2_, 0.1% vol/vol Triton X-100, 1 mM DTT, 100 nM primer, 1 mM dTTP, 1 mM dATP, 10 μM dGTP and 20 μCi [α-32P] dGTP (PerkinElmer). The following primers were used:

Telo 14 5′-biotin-CTAGACCTGTCATCATTAGGGTTAGGGTT-3′;

Telo 16 5′-biotin-CTAGACCTGTCATCATTAGGGTTAGGGTTAG-3′;

Telo 18 5′-biotin-CTAGACCTGTCATCATTAGGGTTAGGGTTAGGG-3′;

Telo 20 5′-biotin-CTAGACCTGTCATCATTAGGGTTAGGGTTAGGGTT.

Telomerase extension was quenched with addition of 200 μl of stop buffer (500 mM NaCl, 10 mM EDTA, 0.1% vol/vol Triton X-100) and transferred to micro-spin columns containing 40 μl Ultralink Neutravidin Plus beads slurry (Thermo Fisher) and rotated at room temperature for 2 h. Non-biotinylated product was removed by centrifugation at 2000 × *g* for 10 s and the beads were washed three times in 500 μl of stop buffer with rotation for 10 min at room temperature followed by centrifugation at 2000 × *g* for 10 s. Residual stop buffer was removed with centrifugation at 2000 × *g* for 10 s. Telomerase extension products were released by addition of 30 μl of denaturing formamide buffer (9 volumes of 90% vol/vol formamide, 10% TBE (90 mM Tris base, 90 mM boric acid, 1 mM EDTA), 0.01% wt/vol xylene cyanol and 0.01% wt/vol bromophenol blue to 1 volume buffer TE containing 5 mM biotin) heating at 95 °C for 10 min. A volume of 5 μl of each DNA sample was analysed by electrophoresis on a DNA sequencing gel run at a constant 75 W for 1 h. The gel was transferred to filter paper, dried for 30 min at 80 °C, exposed to a PhosphorImager screen (GE) and visualised on a Typhoon FLA 9500 scanner.

### Bind-n-Seq analysis of protein–ssDNA specificity

Target sequences of cPPR-Telo1 were determined using a modified version of Bind-n-Seq^48^. An aliquot of 100 nM of purified cPPR-Telo1 was combined with 50 µM ssDNA library oligonucleotide with the sequence: 5′-CTTTATCCAGCCCTCACNNNNNNNNNNCTATAGTGTCACCTAAATC-3′ for 1 h in EMSA buffer (10 mM HEPES, pH 8.0, 1 mM EDTA, 50 mM KCl, 2 mM DTT, 0.1 mg/ml fatty acid-free BSA and 0.02% Tween-20) with gentle rocking at room temperature. An aliquot was removed to serve as the unselected library. His Select Beads (Sigma) were added and the sample was incubated for 30 min at room temperature. Complexes were washed three times with EMSA buffer for 10 min, once with wash buffer (50 mM Tris-HCl, pH 8.0, 0.3 M NaCl and 10 mM imidazole) and proteins were eluted in elution buffer (same as wash buffer but with 250 mM imidazole) for 10 min at room temperature. DNA was purified using Oligo Clean & Concentrator columns following the manufacturer’s instructions (Zymo) and amplified by PCR using the following primers (with Illumina adaptor sequences shown in lower case): 5′-tcgtcggcagcgtcagatgtgtataagagacagCTTTATCCAGCCCTCAC-3′ and 5′-gtctcgtgggctcggagatgtgtataagagacagGATTTAGGTGACACTATAG-3′. PCR products were purified using Oligo Clean & Concentrator columns, following the manufacturer’s instructions (Zymo), indexed and sequenced by the Australian Genomic Research Facility (Perth, Australia) on an Illumina MiSeq, according to the manufacturer’s instructions. Paired-end reads were merged with bbmerge.sh (mininsert = 0 mininsert0 = 0) from the BBTools suite v37.02 (https://jgi.doe.gov/data-and-tools/bbtools/), removing the adapters and the merged reads were trimmed of 5′ and 3′ flanking sequences with cutadapt v1.1.4^49^ (-g ^CTTTATCCAGCCCTCAC -a CTATAGTGTCACCTAAATC$ -n 2). Any trimmed reads not 10 nt in length were excluded using reformat.sh from BBTools (minlength = 10 maxlength = 10) and these sequences searched for 10-nt motifs enriched relative to the control with DREME^50^ (-k 10 -dna -norc).

### Data availability

Crystallography data sets are available at the RCSB Protein Data Bank with accession numbers 5ORM (cPPR-Telo1) and 5ORQ (cPPR-Telo1/ssDNA). The nucleotide sequence for cPPR-Telo1 is available at the NCBI Genbank database with accession code MH247127.

## Electronic supplementary material

Supplementary Information
