# Supplementary material for: Modular ssDNA binding and inhibition of telomerase activity by designer PPR proteins
Source: Nat Commun. 2018 Jun 7;9:2212. doi: 10.1038/s41467-018-04388-1 (PMC5992170; doi:10.1038/s41467-018-04388-1)
Supplement: Supplementary file 1 — Supplementary Information [file 41467_2018_4388_MOESM1_ESM.pdf]

Supplementary Information for

Modular ssDNA binding and inhibition of telomerase activity by designer PPR proteins

Spåhr *et al.*

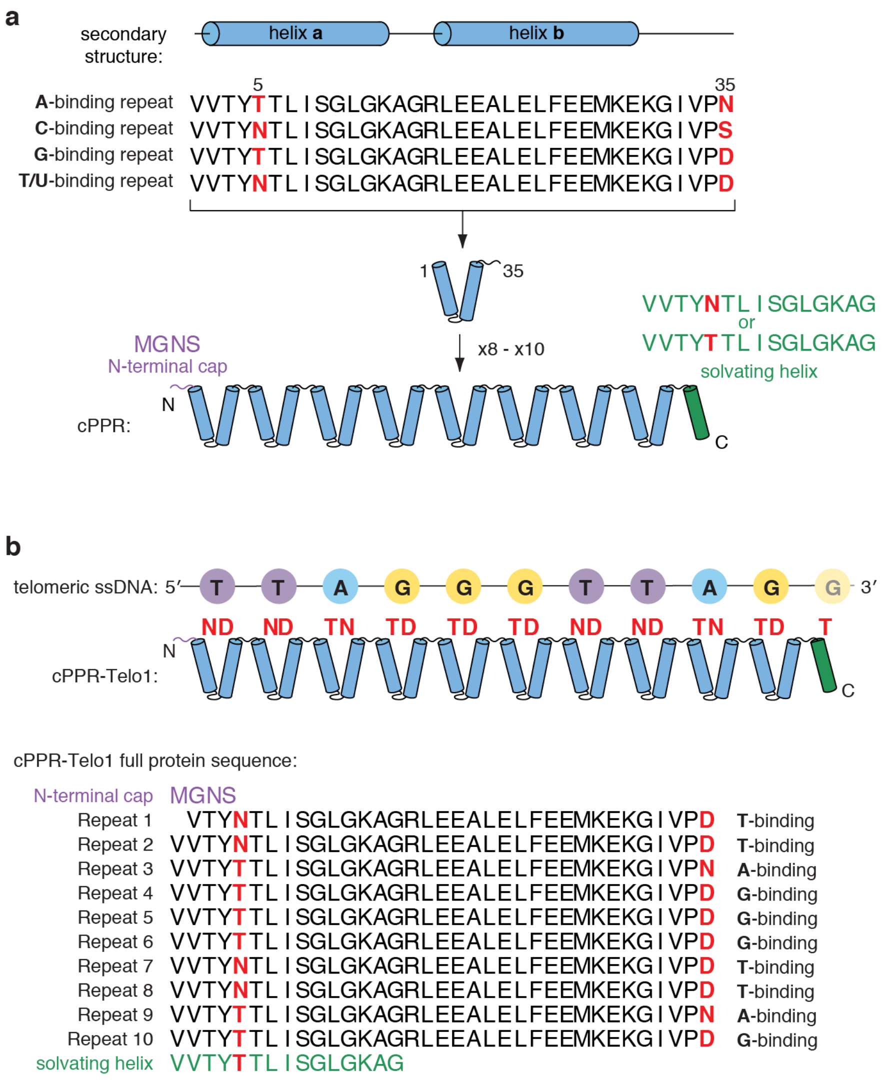

Supplementary Fig. 1 Design rules for cPPR proteins. (**a**) Schematic illustration of the design of cPPR proteins incorporating internal 35-amino acid repeats, where positions 5 and 35 are chosen based on the target ssDNA or RNA base, and flanking N-terminal cap and C-terminal solvating helix stabilise the structure. The identity of the amino acid at position 5 of the C-terminal solvating helix is based on the base 3′ of the target sequence with Asn (N) in the case of a purine and Thr (T) in the case of a pyrimidine. (**b**) An example: the design and protein sequence of cPPR-Telo1, a protein designed to target the telomeric ssDNA sequence 5′-TTAGGGTTAG-3′.

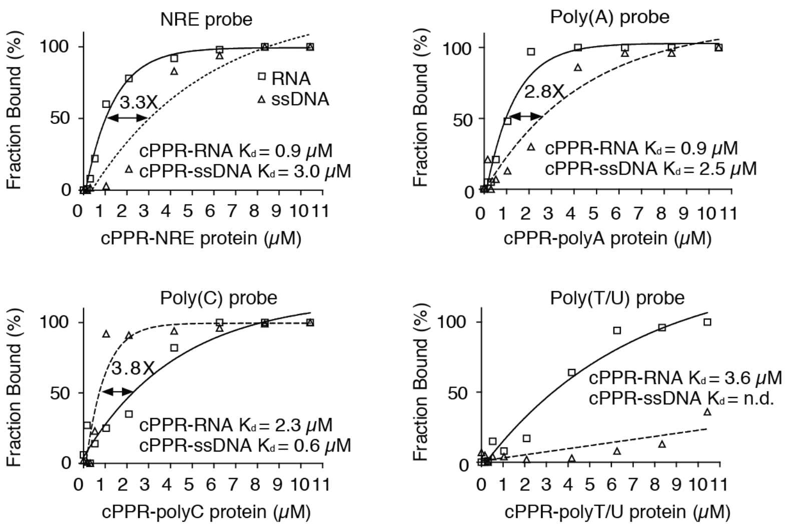

Supplementary Fig. 2 cPPR proteins bind ssDNA and RNA. Equilibrium binding curves for cPPR-NRE, cPPR-polyA, cPPR-polyC and cPPR-polyU/T**.** The lines represent theoretical binding curves fit to the data for cPPR-RNA binding and cPPR-ssDNA binding shown as squares and triangles, respectively. Kd values and the ratio of the Kd are indicated.

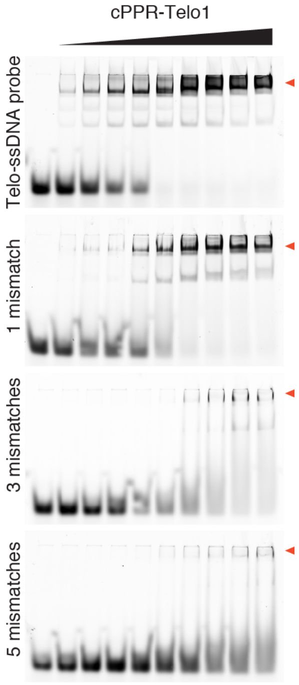

Supplementary Fig. 3 cPPR-Telo1 binding to telomeric ssDNA sequences is highly specific. (**a**) EMSA analyses of cPPR-Telo1 binding to telomeric ssDNA probes with variable numbers of base mismatches. Bound complexes are highlighted with red arrows. Protein concentrations used were, from left to right: 0, 0.15 μM, 0.3 μM, 0.5 μM, 1 μM, 2 μM, 4 μM, 6 μM, 8 μM, 10 μM.

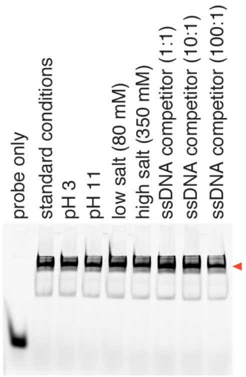

Supplementary Fig. 4 Robust binding of ssDNA in the presence of variable salt concentrations, pH, and competitor ssDNA. The standard EMSA reaction contain 5 μM cPPR-Telo1, 0.42 μM telomeric ssDNA probe, 105 mM salt, pH 7. Test reactions have altered buffers with low salt (80 mM), high salt (350 mM), pH 3, pH 11, and increasing amounts of competitor ssDNA (1:1, 10:1, and 100:1 ratios of competitor to telomeric DNA probe). Bound complexes are highlighted with a red arrow.

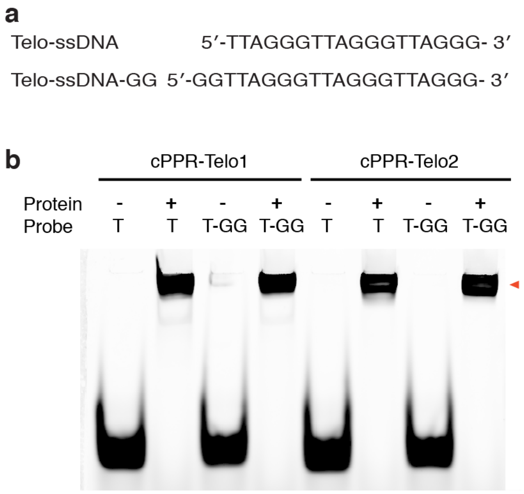

Supplementary Fig. 5 cPPR proteins bind ssDNA independent of potential G-quadruplexes. (**a**) Sequences of Telo-ssDNA and Telo-ssDNA-GG. Telo-ssDNA-GG is the same as Telo-ssDNA except that it contains two additional G nucleotides at the 5′-end. (**b**) Native gel analysis of 100 nM ssDNA in the presence (+) or absence (-) of 333 nM cPPR-Telo1 or cPPR-Telo2. Bound complexes are highlighted with a red arrow.

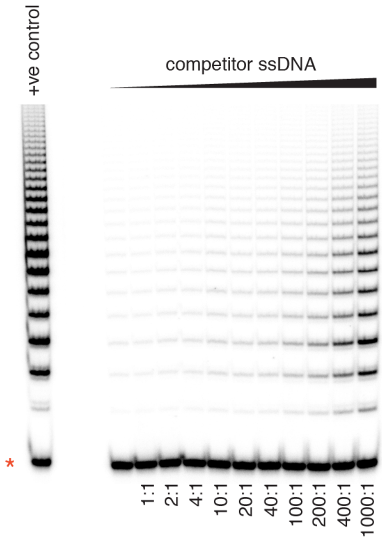

Supplementary Fig. 6 Robust inhibition of telomerase activity by cPPR-Telo1 in the presence of competitor ssDNA. A non-specific competitor ssDNA derived from the 15-nt of spacer DNA of the telomerase extension assay substrate was added in increasing ratios relative to telomeric ssDNA substrate. The red asterisk indicates a 30-mer 5′-^{32}P-labelled recovery/loading control.

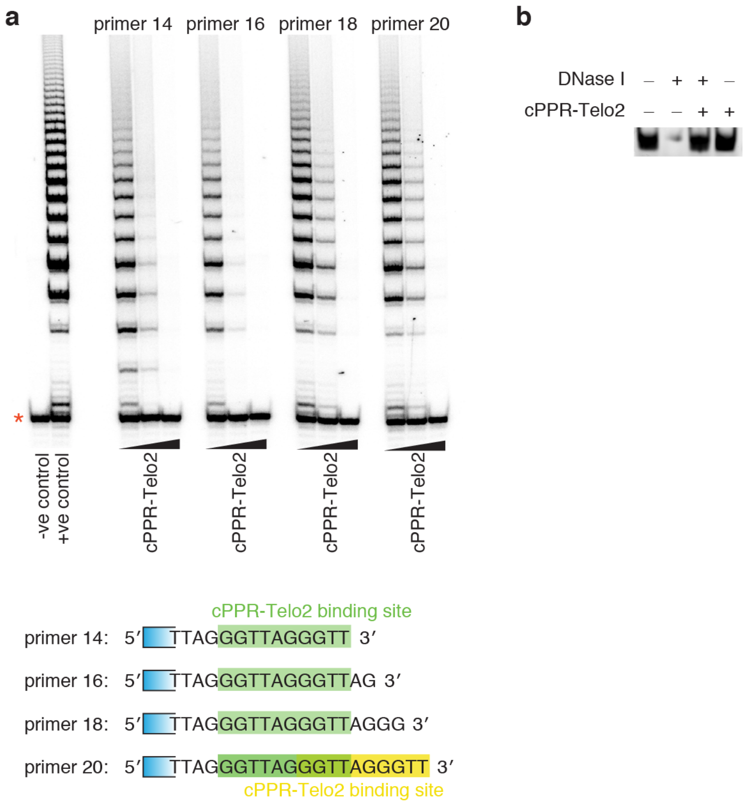

Supplementary Fig. 7 Mode of telomerase inhibition by cPPR proteins. (**a**) Telomerase activity assays with primers incorporating varying lengths of telomeric sequence and increasing concentrations of cPPR-Telo2 (200 nM and 300 nM). The red asterisk indicates a 30-mer 5′-^{32}P-labelled recovery/loading control. (**b**) cPPR-Telo2 protects its ssDNA target from DNase I digestion.

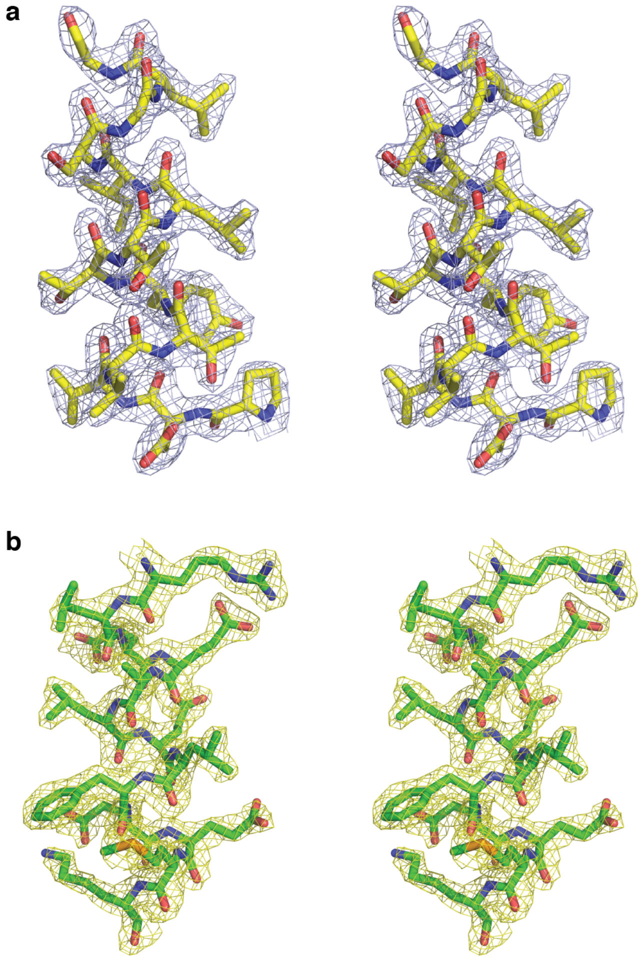

Supplementary Fig. 8 Stereo images of the electron density of cPPR-Telo1. (**a**) Stereo image showing the final weighted $2F_O-F_C$ electron density map of a representative section of cPPR-Telo1 contoured at 1.6 σ. Residues are shown as yellow sticks and map in blue mesh. (**b**) Stereo image showing the final weighted $2F_O-F_C$ electron density map of a representative section of cPPR-Telo1 in complex with DNA contoured at 1.6 σ. Residues are shown as green sticks and map in yellow mesh.

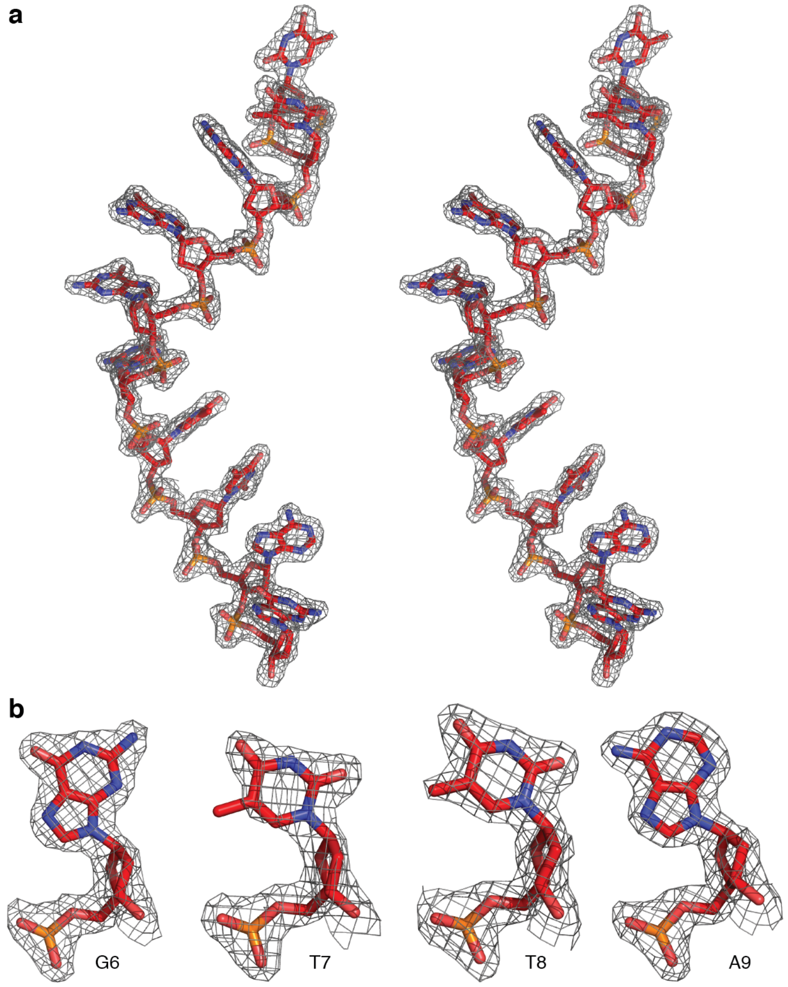

Supplementary Fig. 9 Electron density of the cPPR-bound ssDNA. (**a**) Stereo image showing the final weighted $2F_O$-F_C electron density map of the DNA contoured at 1.6 σ. Residues are shown as red sticks and map in grey mesh. (**b**) Guanine (G), thymine (T), and adenine (A) nucleobases shown as red sticks and the final weighted $2F_O$-F_C electron density map contoured at 1.6 σ in grey mesh.

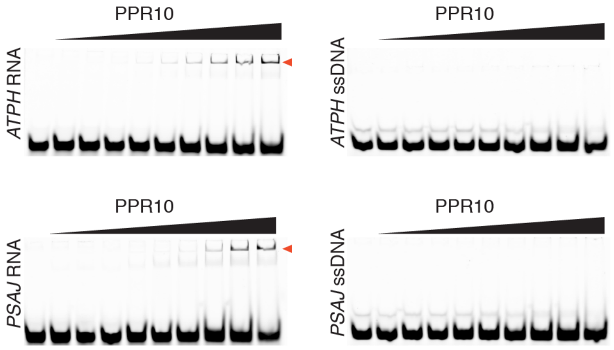

Supplementary Fig. 10 PPR10 does not bind ssDNA. EMSA analyses of PPR10 binding of *PSAJ* and *ATPH* ssDNA and RNA probes. Bound complexes are highlighted with red arrows.
